# Prevalence of exclusive breastfeeding for the first six months of an infant’s life and associated factors in a low–middle income country

**DOI:** 10.1186/s13006-023-00585-x

**Published:** 2023-08-31

**Authors:** Hoang Thi Nam Giang, Do Thi Thuy Duy, Nguyen Lam Vuong, Nguyen Thi Tu Ngoc, Thu Thi Pham, Nguyen Tran Minh Duc, Trinh Thi Diem Le, Tran Thi Tuyet Nga, Le Tho Minh Hieu, Nguyen Thi Tuong Vi, Bui Minh Triet, Nguyen Tan Thach, Tran Thi Bach Truc, Nguyen Tien Huy

**Affiliations:** 1 School of Medicine and Pharmacy, The University of Danang, Danang, Vietnam; 2 University of Medicine and Pharmacy at Ho Chi Minh City, Ho Chi Minh City, Vietnam; 3 Thai Nguyen University of Medicine and Pharmacy, Thai Nguyen, Vietnam; 4 School of Tropical Medicine and Global Health, Nagasaki University, Nagasaki, Japan

**Keywords:** Breastfeeding, Exclusive breastfeeding, Associated factors, Low–middle income country, Skin-to-skin contact, Early initiation of breastfeeding, Breastfeeding intention, “dose-response” relationship

## Abstract

**Background:**

Although breastfeeding is practiced by 98% of mothers in Vietnam, infant breastfeeding behaviors remain far from World Health Organization recommendations and continues to decline. This study aims to explore the prevalence and factors associated with exclusive breastfeeding in the first six months of an infant’s life.

**Methods:**

A cross-sectional study utilized a self-administered maternal questionnaire to collect data on 1072 Vietnamese mothers who brought infants aged between 6 and 30 months to a community health centre (CHC) for routine vaccination. Data collection was conducted from March to May 2021 in two cities in Central and North Vietnam. In order to measure exclusive breastfeeding, we asked mothers to recall (yes / no), if the child had received breast milk, formula, colostrum milk powder, water, vitamin / medicine, fruit juice / honey, and complementary foods aged under six months.

**Results:**

In the first six months, 14.2% of mothers exclusively breastfed their infants. Multivariable logistic regression analysis demonstrated a significant association between exclusive infant breastfeeding and the highest maternal education level (university or postgraduate) (adjusted odds ratio (aOR) 2.55; 95% confidence interval (CI) 1.10, 5.91); male infants (aOR 1.72; 95% CI 1.11, 2.68); duration of skin-to-skin contact greater than 90 min (aOR 7.69; 95% CI 1.95, 30.38); receiving first breastfeeding during skin-to-skin contact (aOR 2.31; 95% CI 1.30, 4.10); completely feeding infant directly at the breast (aOR 1.65; 95% CI 1.00, 2.71) and exclusive breastfeeding intention during pregnancy (aOR 2.48; 95% CI 1.53, 4.00). When compared with mothers who were prenatally exposed to infant formula advertising classified as “often”, the prevalence of exclusive infant breastfeeding was higher in mothers who classified their prenatal exposure to infant formula advertising as “sometimes” (aOR 2.15; 95% CI 1.13, 4.10), and “seldom” (aOR 2.58; 95% CI 1.25, 5.36).

**Conclusion:**

The prevalence of mothers who practiced exclusive infant breastfeeding during the first six months in Vietnam was low. Infants should receive early maternal-infant skin-to-skin contact greater than 90 min and complete first breastfeeding during skin-to-skin contact. Further, mothers should be protected against infant formula advertisements to maximise the likelihood of exclusive breastfeeding during the child’s infancy.

## Background

Despite significant evidence of the benefits of breastfeeding, nearly 60% of infants younger than six months are not exclusively breastfed worldwide – a proportion that has had minimal improvement over the past two decades [1]. In low-income countries, 53% of infants under six months were not exclusively breastfed, while these rates were 61% and 63% in lower-middle income and upper-middle income countries respectively [2]. Further, middle-income and high-income countries tend to have a shorter duration of infant breastfeeding compared to low-income countries [2]. In May 2012, the World Health Assembly Resolution set Goal 65.6 with the target that at least 50% of infants should be exclusively breastfed for the first six months of life by 2025 [3]. Further, they found that stronger commitment and much more effort from governments and health authorities are in urgent need to achieve this breastfeeding goal.

The determinants of breastfeeding are multifactorial. They range from medical disorders, hospital practices, socioeconomic factors, cultural attitudes, media and marketing, workplace support, to individual characteristics [4]. Nearly all maternal-infant dyads can breastfeed, with few maternal or infant conditions requiring avoidance of breastfeeding such as galactosemia [5]. Health workers can influence feeding decisions at important moments such as right after birth and support breastfeeding efforts when challenges occur. Conversely, early mother-infant separation, and the recommendation to use breast milk substitutes during the maternal hospitalization are hospital practices that negatively impact breastfeeding [6]. Additionally, the availability of infant formula also limits breastfeeding [7]. Further, aggressive marketing of infant formula has been found to be associated with discontinuation of exclusive infant breastfeeding [8] and impacts maternal decisions on infant feeding practices [9]. Besides maternal lack of awareness, in some traditional cultural beliefs, colostrum is considered harmful to infants and is discarded instead of being used [10]. Finally, the leading reason for early weaning of breastfeeding can be attributed to women’s need to return to the workplace soon after childbirth. A longer duration of postpartum maternity leave was associated with an increased prevalence of long duration of breastfeeding [11].

Vietnam is a low–middle income country in the World Health Organization (WHO) Western Pacific Region. Since the early 1990s, Vietnam has implemented national initiatives to create a strong regulatory environment to protect, promote and support infant breastfeeding [7]. The Baby Friendly Hospital Initiative (BFHI) was implemented in Vietnam in 1992 with the goal of adhering to the WHO’s Ten Steps to Successful Breastfeeding [12]. Since 2013, Vietnamese mothers with newborn infants have been entitled to six months of maternity leave after delivery which is similar to the WHO recommendation of approximately six months of exclusive infant breastfeeding. In 2014, early essential newborn care (EENC) was adopted in Vietnam which led to improvements in early initiation of breastfeeding [13]. In the same year, the Prime Minister approved the Vietnam Government Decree 100, which imposed regulations on the marketing and use of nutritional products for young children, feeding bottles, teats, and pacifiers [14]. Despite these successive national efforts, the proportion of mothers breastfeeding in Vietnam is far from WHO recommendations and continues to decline [15, 16]. Although breastfeeding is practiced by 98% of mothers in Vietnam, infant breastfeeding behaviors remains far from ideal [17]. Results from the Multiple Indicator Cluster Surveys in Vietnam between 2000 and 2011 showed that exclusive breastfeeding for the first six months decreased by 8% between 2000 (25%) and 2006 (17%), and remained unchanged between 2006 (17%) and 2011 (17%) [15]. Another study conducted in southern Vietnam reported the prevalence of exclusive infant breastfeeding at 33% before hospital discharge, but this declined to 15% at four months and less than 1% at six months of life [16]. Mothers whose infants were delivered by Cesarean sections were also associated with delayed breastfeeding initiation, which is an important predictor of continued and exclusive infant breastfeeding [18]. In Vietnam, Cesarean section rates have increased significantly in recent years, with above 50% of infants being delivered by Cesarean section in some cities, complicating efforts to improve infant breastfeeding practices [19, 20]. Other factors associated with breastfeeding practices in Vietnam included socioeconomic status, mother’s employment outside of the home, lack of breastfeeding knowledge or misinformation, mother’s highest education level, father’s occupation, and infant feeding preferences of the father [21, 22]. Prior to this study conducted in two cities in North and Central Vietnam, however, there have been few studies published regarding the prevalence of exclusive infant breastfeeding for the first six months which include Vietnam’s EENC and level of mother’s exposure to infant formula advertising as potential related factors for infant breastfeeding. In addition, these studies’ relevance to the Vietnamese infant population may be limited due to small sample sizes. Thus, the objective of this current study is to determine the prevalence of six-month breastfeeding and the associated factors which influence maternal infant breastfeeding practices.

## Methods

### Study design

A cross-sectional study utilized a designed questionnaire that was given to mothers to collect data from March to May 2021. One core member of the research team in each city was responsible for supervising data collection. Each core member then trained and supervised all data collectors and routinely checked all completed maternal questionnaires. Data collectors at each site were responsible for distributing questionnaires to eligible mothers and collecting completed questionnaires.

### Study setting

Vietnam is the third most populous country in Southeast Asia with over 97 million people in 2020, with nearly one-third of that population living in rural areas. In 2019, there were 24.9 million women of reproductive age (15–49 years) and approximately 1.6 million live births. At that time, the mortality rate for children under the age of 5 was 20 per 1000 live births, the infant mortality rate was 16 per 1000 live births, and the neonatal mortality was 10 per 1000 live births [23].

This study was conducted in two cities in Central and North Vietnam. The first city, Da Nang, is one of the most important port cities in Central Vietnam with a population of over 1.1 million in 2020. The city has a total natural land area of around 1283 km^2^, of which 241 km^2^ are urban districts and it has the highest urbanization ratio among provinces in Vietnam, with nearly 90% of the population living in urban areas. The neonatal mortality in Da Nang was 4 per 1000 live births. The second study city, Thai Nguyen City, is located in the centre of the Northern region which is close to Ha Noi, the capital city. Thai Nguyen City has a natural land area of about 223 km^2^ and a population of approximately 340,000 in 2019. In total, there are 32 CHCs in Thai Nguyen City and 56 CHCs in Da Nang City.

There were eight selected sites (CHC) in Thai Nguyen City. Four of the selected CHCs were located in communities categorized as urban areas and four CHCs were in areas categorized as semi-urban (ward) areas. In Da Nang City, ten CHCs were selected and were located in the urban areas and six CHCs were located in the surrounding semi-urban communities. Our sampling method was based on our ability to get data collection approval from the CHCs as the study was conducted during the COVID-19 pandemic.

### Participants

Mothers whose infants were aged greater than 6 months to 30 months who presented to the CHCs for routine vaccination were invited to join the study. In Vietnam, the national Expanded Programme on Immunization is integrated into the services of CHCs. All children in Vietnam are required to be fully immunized according to the Expanded Programme on Immunization. The pediatric vaccination participation rate is greater than 95% in the country. Only mothers of singletons were included. After maternal verbal informed consent was given to participate in the study, each mother received a self-administered questionnaire.

### Questionnaire and data collection

Our study questionnaire was based both on a literature review of breastfeeding patterns [16, 24–27] and from consulting with maternal-infant clinical specialists who have experience in infant breastfeeding and EENC practices. The full questionnaire consisted of 39 self-administered questions to each mother and the questions asked were to help determine potential factors associated with breastfeeding in the first six months of the infants life including: (1) maternal socio-demographic information (age, education level); (2) place of residence; (3) parity; (4) age of infant when mother returned to work after maternity leave; (5) complications during pregnancy and at delivery; (6) infant characteristics (sex, birth weight, mode of delivery, hospital of birth, complication at birth); (7) skin-to-skin contact practice after birth (no, < 15 min, 15–90 min, > 90 min); (8) having first breastfeed during skin-to-skin contact; (9) breastfeeding methods (direct or mixed between direct and expressed breast milk); (10) breastfeeding difficulties; 11) level of exposure to infant formula advertising during pregnancy (“often”, “sometimes”, “seldom”); 12) receiving breastfeeding counselling; 13) intention of feeding pattern during pregnancy; 14) knowledge on definition and duration of exclusive breastfeeding; and 15) breastfeeding support in the workplace. These variables were chosen based on the review of existing publications and current practice on EENC in Vietnam [13, 21, 25–27]. Our study questionnaire has not been previously utilized or published.

The cut-off values of the duration of skin-to-skin contact were based on the dividing values to assess how long after birth the baby received first breastfeeding in the WHO checklist used to interview postpartum mothers [28]. A draft questionnaire was sent to ten mothers to test for confusion about any questions and were then asked for any suggestions or changes to the draft questionnaire. After receiving feedback from the initial pilot testing, the questionnaire was revised, and questions were rearranged for better clarity when being answered.

The primary outcome of interest was the prevalence of exclusive infant breastfeeding for the first six months of life. As defined by the WHO, exclusive infant breastfeeding is an infant being fed only breast milk as the sole source of nutrition. No other liquids, solids, or water is consumed by the infants, except vitamins, minerals, and medicines [29]. Exclusive infant breastfeeding can either be direct breastfeeding or expressed breast milk feeding. In our study, we asked mothers what their infant’s primary source of nutrition was when they were under six months of age with the options including breast milk (yes / no), infant formula (yes / no), colostrum milk powder (yes / no). Other supplements were also asked about including water (yes / no), vitamin / medicine (yes / no), fruit juice / honey (yes / no), and complementary foods (yes / no). Exclusive breastfeeding was identified based on the answer “yes” for the breast milk and “no” for infant formula, colostrum milk powder, water, fruit juice / honey, and complementary foods.

Printed questionnaires were distributed to eligible mothers while at the CHCs. For mothers who were holding their infants and required help, the data collectors read the questionnaire and recorded their answers. The collectors sat down in the same direction as the mothers so that they could still view the questionnaire. Our study followed A Consensus-Based Checklist for Reporting of Survey Studies (CROSS) [30].

### Sampling technique and sample size determination

**Fig. 1:**
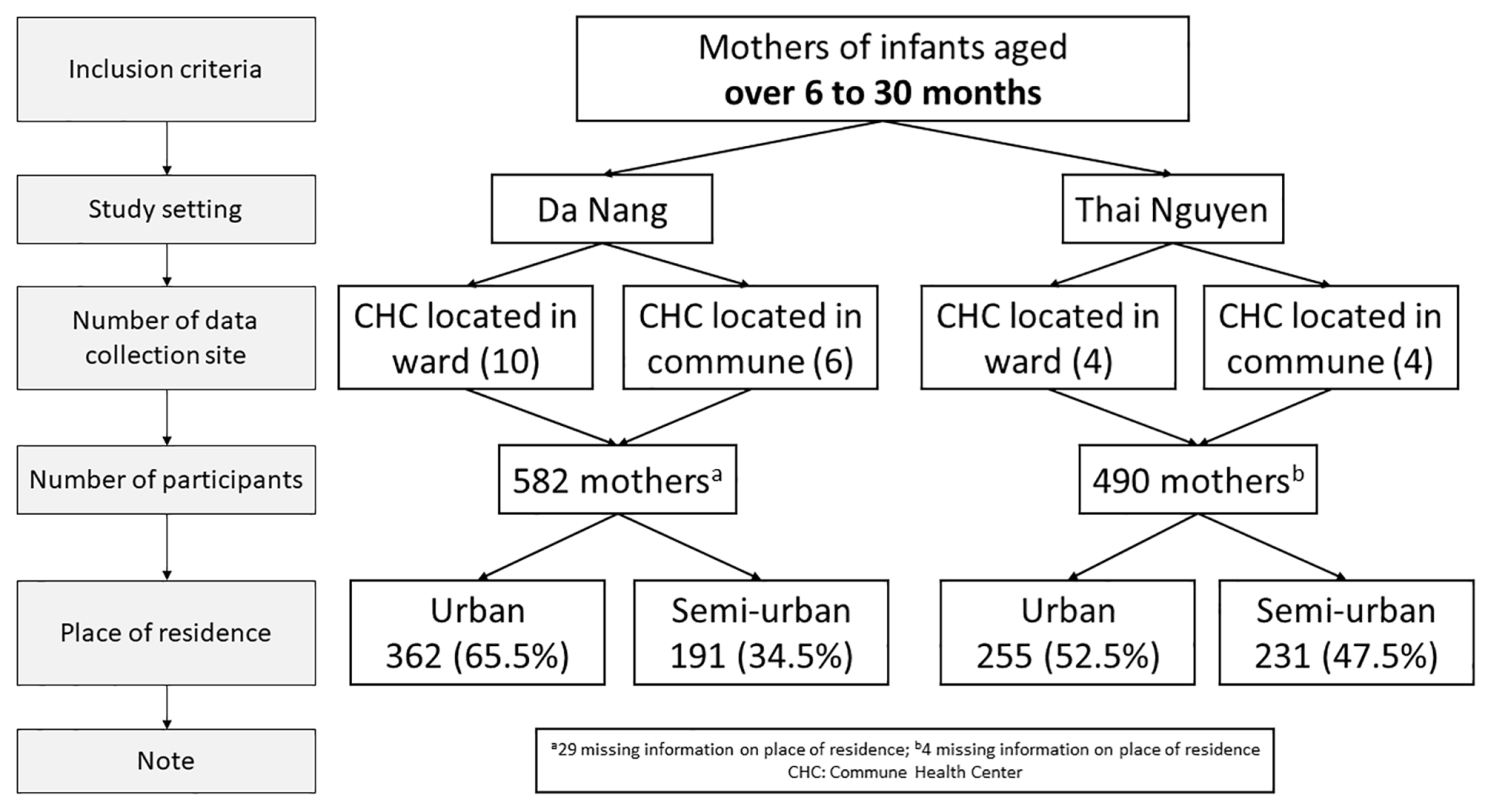
Sampling procedure and study participants

The sample size was calculated by using the formula of estimating prevalence with the following assumptions: Prevalence of exclusive breastfeeding was 19.6% which was taken from the National Institute of Nutrition [31], with 95% confidence interval, precision error of 2.5%, and 5% non-response rate. The required sample size was 1018 participants. Finally, a total of 1072 mothers were included in the analysis. The detailed sampling procedure is described in Fig. 1.

### Data quality control and data analysis

All completed questionnaires were carefully checked before entering data in EpiData version 3.1. Data entry forms were designed to minimize missing and incorrect data such as value ranges and dropdown lists.

Descriptive analysis was done for all variables in the study. Categorical variables were presented with frequency and percentage. Continuous variables were presented with mean and standard deviation (SD) or median and interquartile range (IQR). The prevalence of exclusive breastfeeding for the first six months is defined as percentage of infants who were fed exclusively with breast milk when they were less than six months as reported by mothers.

Binary logistic regression analysis was used to estimate the crude odds ratio (OR) and 95% confidence interval (CI) between the outcome (exclusive breastfeeding) and independent variables. Multivariable logistic regression was used to explore factors associated with the outcome and reported using adjusted OR (aOR) and 95% CI. A *P*-value of < 0.05 was considered statistically significant. All analyses were done using R version 3.6.1.

### Ethics approval and informed consent

The study was conducted according to the guidelines of the Declaration of Helsinki, and approved by the Institutional Review Board of The University of Medicine and Pharmacy at Ho Chi Minh City according to Decision No. 990 / HĐĐĐ-ĐHYD date 11 / 01 / 2020. The surveys were anonymous to protect the identities of both the mother and infant.

## Results

### Maternal and infant characteristics

A total of 1072 mothers of infants aged over 6 to 30 months completed questionnaires. The background characteristics of mothers and their infants are presented in Table 1. Mothers’ age ranged from 18 to 46 with a mean of 29.8 ± 4.9. The majority of the mothers (87.1%) had an education level of high school or higher.

**Table 1:** Characteristic of mothers, infants, skin-to-skin contact practices and breastfeeding practices, Vietnam, 2021 (n = 1072)

Characteristic	Summary statistics n (%)
**Maternal characteristics**	
Age, years, mean ± SD	29.8 ± 4.9
Education qualification (n = 1068)	
- Primary school & Secondary school	138 (12.9)
- High school/intermediate degree	586 (54.9)
- University/postgraduate	344 (32.2)
Place of residence	
- Urban	617 (59.4)
- Semi-urban	422 (40.6)
Parity, (mean ± SD)	1.8 ± 0.75
- 1	339 (31.7)
- 2	536 (50.0)
- ≥ 3	196 (18.3)
Complication during pregnancy and at birth (Yes)	96 (0.9)
**Infant characteristics**	
Sex, female	524 (49.0)
Birth weight (Kg), mean ± SD	3.2 (0.4)
Cesarean section	539 (50.4)
Hospital of birth	
- Central/Provincial	728 (68.4)
- District	242 (22.7)
- Private	86 (8.1)
- Commune	8 (0.8)
Complication at birth	59 (5.6)
- Required resuscitation in delivery room	35 (3.3)
- Admission to NICU	24 (2.3)
**Skin-to-skin contact practice**	
Duration of skin-to-skin contact	
- None	136 (12.7)
- < 15 min	315 (29.5)
- 15–90 min	428 (40.1)
- > 90 min	189 (17.7)
Had a first breastfeed during skin-to-skin contact	499 (53.3)
The way of breastfeeding infants	
- Direct breastfeeding only	655 (61.1)
- Mixed feeding (direct and expressed breast milk)	417 (38.9)
Breastfeeding problem (Yes)	174 (16.3)
Exposed to formula advertising during pregnancy	
- Often	235 (23.6)
- Sometimes	551 (51.5)
- Seldom	266 (24.9)
Received breastfeeding counselling (Yes)	795 (74.2)
Intention of feeding pattern during pregnancy	795 (74.2)
- Exclusive breastfeeding	587 (54.8)
- Combination of breast milk and formula	468 (43.7)
- Exclusive formula	17 (1.5)
Correctly defined exclusive breastfeeding	665 (62.1)
Correctly identified recommended duration of exclusive breastfeeding	550 (51.3)
Breastfeeding support in the workplace (Yes)	340 (35.9)
**Breastfeeding practices for the first 6 months of life**	
- Exclusive breastfeeding	152 (14.2)
- Breastfeeding	1055 (99.3)
- Formula	683 (63.7)
- Colostrum milk powder	209 (19.5)
- Vitamin/ Medicines	466 (43.5)
- Water	391 (36.5)
- Fruit juices/ Honey	138 (12.9)
- Complementary feeding	454 (42.4)

SD (standard deviation); IQR (interquartile range); NICU (neonatal intensive care unit)

Approximately half of the study infants (50.4%) were born by Cesarean section. A majority of the mothers (87.3%) reported they had skin-to-skin contact with their infants after birth, but only 17.7% of mothers experienced more than 90 min of skin-to-skin contact with their infants, and 53.3% of infants had first breastfeeding during skin-to-skin contact.

A majority of the mothers (74.2%) received breastfeeding counselling; 62.1% of mothers were able to correctly define exclusive breastfeeding; and 51.3% correctly identify recommended duration of exclusive breastfeeding. During pregnancy, 54.8% of the mothers stated an intention to breastfeed their babies exclusively for the first six months of life, and the remaining stated an intention to feed the infants either with the combination of breast milk and infant formula (43.7%) or with formula only (1.5%). Furthermore, exposure to infant formula advertising during pregnancy was reported as “often” in 23.6% of mothers, “sometimes” in 51.5% of mothers, and “seldom” in 24.9% of mothers.

### Breastfeeding practice

In our study, nearly all the infants were breastfed (99.3%) with the prevalence of exclusive infant breastfeeding in the first six months of life at 14.2%. Approximately one-third of the mothers (38.9%) practice mixed feeding (a combination of direct breast feeding and expressed breast milk). In non-breastfed infants, 63.7% were given infant formula and 42.2% received complementary food feedings when they were less than six months of age (Table 1).

**Fig. 2:**
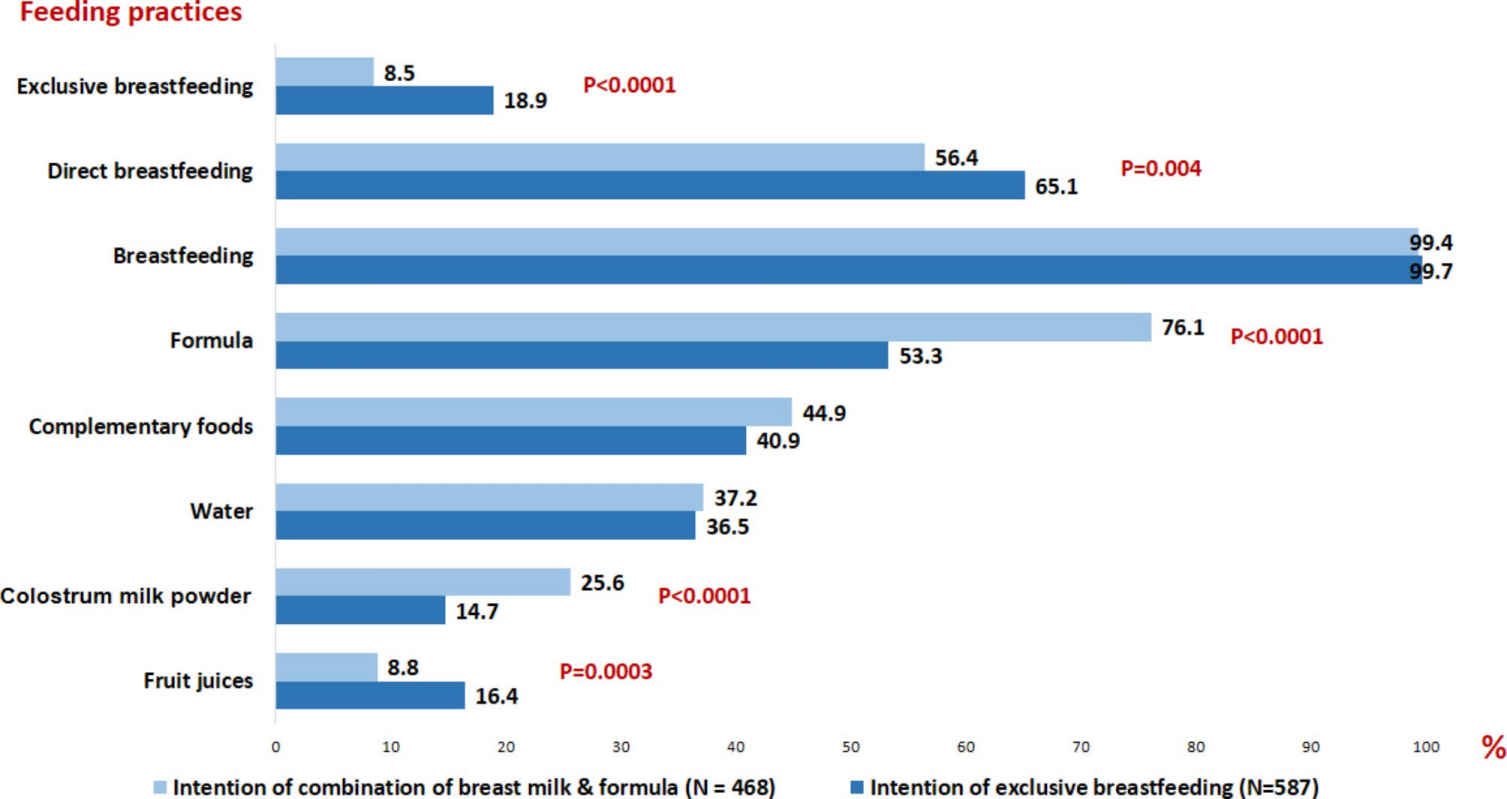
Differences in feeding practices between mothers who had prenatal intention to exclusively breastfeed and mothers who had prenatal intention to combine breast milk and formula

Differences in feeding practices between mothers who had prenatal intention to exclusively breastfeed and mothers who had prenatal intention to combine breast milk and infant formula are presented in Fig. 2. The exclusive breastfeeding proportion was significantly higher in mothers who intended to breastfeed their babies exclusively compared to those who intended to feed the infants a combination of breast milk and infant formula (*p* < 0.0001).

### Factors associated with exclusive breastfeeding for the first six months of life

In multivariable logistic regression analysis, level of education; sex of infants (male); duration of skin-to-skin contact; having first breastfeeding during skin-to-skin contact; feeding infant directly at the breast exclusively; level of exposure to infant formula advertising; and exclusive breastfeeding intention during pregnancy were significant predictors of exclusive infant breastfeeding (Table 2). For exclusive breastfeeding the aOR was 2.55 (95% CI 1.10, 5.91) times higher among mothers who had a university or postgraduate degree than among mothers with a primary and secondary school level of education. Mothers who experienced skin-to-skin contact had higher odds of exclusive breastfeeding compared to mothers who did not experience skin-to-skin contact. A longer duration of skin-to-skin contact in our study was also associated with higher odds of exclusive breastfeeding. The aORs of exclusive breastfeeding in the group with skin-to-skin contact were 7.69 (95% CI 1.95, 30.38), 3.64 (95% CI 0.98, 13.59), and 1.83 (95% CI 0.49, 6.78) times higher for skin-to-skin contact of > 90 min, 15─90 min, and < 15 min respectively compared to the group without skin-to-skin contact. Further, infants who completed their first breastfeeding while having skin-to-skin contact had a positive effect on exclusive breastfeeding (aOR 2.31; 95% CI 1.30, 4.10) and male infants were more likely to be exclusively breastfed compared to female infants (aOR 1.72; 95% CI 1.11, 2.68). The mothers who had a prenatal intention to exclusively breastfeed had 2.48 times the aOR of exclusive breastfeeding for the first six months of life compared to the mothers who had a prenatal intention to combine breast milk and infant formula feeding (95% CI 1.53, 4.00). The level of exposure to infant formula advertising during pregnancy showed a “dose-response” type relationship. Mothers who classified their exposure to infant formula advertising as “sometimes” were associated with aOR 2.15; 95% CI 1.13, 4.10) times higher odds of exclusive breastfeeding compared to those whose exposure to advertising was classified as “often”. Mothers who classified their exposure to infant formula advertising as “seldom” were associated with aOR 2.58; 95% CI 1.25, 5.36] times higher odds of exclusive infant breastfeeding as compared to those whose exposure to advertising was classified as “often”.

**Table 2:** Factors associated to exclusive breastfeeding for the first 6 months of life (N = 1072)

Characteristics	Non-exclusive (n = 920)	Exclusive (n = 152)	Unadjusted OR (95% CI)	P value	Adjusted OR (95% CI)	P value
Age of mother, mean (SD)	30.6 (4.3)	29.7 (5.0)	1.04 (1.00, 1.07)	0.043	1.00 (0.94, 1.06)	0.934
Mother education level						
- Primary & Secondary	121 (87.7)	17 (12.3)	Reference		Reference	
- High school/ Intermediate degree	515 (87.9)	71 (12.1)	0.98 (0.56, 1.73)	0.948	1.39 (0.63, 3.05)	0.418
- University/ Postgraduate	280 (81.4)	64 (18.6)	1.63 (0.91, 2.89)	0.098	2.55 (1.10, 5.91)	0.029
Complication during pregnancy & at birth						
- No	840 (86.1)	136 (13.9)	Reference		Reference	
- Yes	80 (83.3)	16 (16.7)	1.24 (0.70, 2.18)	0.473	0.84 (0.40, 1.77)	0.646
Place of residence						
- Urban	525 (85.1)	92 (14.9)	Reference		Reference	
- Semi-urban	368 (87.2)	54 (12.8)	0.84 (0.58, 1.20)	0.333	0.89 (0.55, 1.42)	0.614
Parity	2.0 (0.7)	1.8 (0.7)	1.32 (1.06, 1.65)	0.014	1.16 (0.80, 1.67)	0.434
Mode of delivery						
- Cesarean section	464 (86.1)	75 (13.9)	Reference		Reference	
- Vaginal delivery	454 (85.5)	77 (14.5)	1.05 (0.74, 1.48)	0.784	1.03 (0.65, 1.62)	0.908
Hospital of birth						
- Central/Provincial	634 (87.1)	94 (12.9)	Reference		Reference	
- District	199 (82.2)	43 (17.8)	1.46 (0.98, 2.16)	0.061	0.93 (0.54, 1.60)	0.788
- Private	72 (83.7)	14 (16.3)	1.31 (0.71, 2.42)	0.385	1.01 (0.46, 2.24)	0.979
Infant’s sex (male)						
- Female	442 (84.4)	82 (15.6)	Reference		Reference	
- Male	475 (87.2)	70 (12.8)	1.26 (0.89, 1.78)	0.189	1.72 (1.11, 2.68)	0.016
Infant’s birth weight	3.2 (0.4)	3.3 (0.4)	1.52 (1.00, 2.33)	0.051	1.71 (0.98, 2.99)	0.06
Infant’s complication at birth						
- No	857 (85.5)	145 (14.5)	Reference		Reference	
- Yes	54 (91.5)	5 (8.5)	0.55 (0.22, 1.39)	0.171	0.71 (0.16, 3.21)	0.656
Skin-to-skin contact practice (SSC)						
- No SSC	131 (96.3)	5 (3.7)	Reference		Reference	
- < 15 min SSC	293 (93.0)	22 (7.0)	1.97 (0.73, 5.31)	0.181	1.83 (0.49, 6.78)	0.369
- 15–90 min SSC	359 (83.9)	69 (16.1)	5.04 (1.99, 12.75)	< 0.001	3.64 (0.98, 13.59)	0.054
- > 90 min SSC	133 (70.4)	56 (29.6)	11.03 (4.28, 28.4)	< 0.001	7.69 (1.95, 30.38)	0.004
First breastfeeding during SSC						
- No	537 (93.7)	36 (6.3)	Reference		Reference	
- Yes	383 (76.8)	116 (23.2)	4.52 (3.04, 6.71)	< 0.001	2.31 (1.30, 4.10)	0.004
Methods of breastfeeding infants						
- Mixed feeding (direct & expressed breast milk)	380 (91.1)	37 (8.9)	Reference		Reference	
- Direct breastfeeding only	540 (82.4)	115 (17.6)	2.19 (1.48, 3.24)	< 0.001	1.65 (1.00, 2.71)	0.049
Breastfeeding problems						
- No	766 (85.7)	128 (14.3)	Reference		Reference	
- Yes	151 (86.8)	23 (13.2)	0.91 (0.57, 1.47)	0.701	1.12 (0.61, 2.07)	0.713
Exposed to formula advertising during pregnancy						
- Often	227 (89.7)	26 (10.3)	Reference		Reference	
- Sometimes	477 (86.6)	74 (13.4)	1.35 (0.84, 2.18)	0.210	2.15 (1.13, 4.10)	0.020
- Seldom	214 (80.5)	52 (19.5)	2.12 (1.28, 3.52)	0.004	2.58 (1.25, 5.36)	0.011
Received breastfeeding counselling						
- No	237 (85.6)	40 (14.4)	Reference		Reference	
- Yes	683 (85.9)	112 (14.1)	0.97 (0.66, 1.44)	0.885	0.86 (0.52, 1.44)	0.566
Intention of feeding pattern during pregnancy						
- Combination of breast milk & formula	428 (91.5)	40 (8.5)	Reference		Reference	
- Exclusive breastfeeding	476 (81.1)	111 (18.9)	2.50 (1.70, 3.66)	< 0.001	2.48 (1.53, 4.00)	< 0.001
Correctly defined exclusive breastfeeding						
- No	464 (88.9)	58 (11.1)	Reference		Reference	
- Yes	456 (82.9)	94 (17.1)	1.65 (1.16, 2.34)	0.005	1.38 (0.87, 2.19)	0.176
Correctly identified recommended duration of exclusive breastfeeding						
- No	347 (85.5)	59 (14.5)	Reference		Reference	
- Yes	572 (86.0)	93 (14.0)	0.96 (0.67, 1.36)	0.804	0.88 (0.56, 1.40)	0.596
Breastfeeding support in the workplace						
- No	533 (88.0)	73 (12.0)	Reference		Reference	
- Yes	284 (83.5)	56 (15.6)	1.44 (0.99, 2.10)	0.058	1.38 (0.88, 2.17)	0.164

SD: standard deviation; SSC: skin-to-skin contact; OR: odds ratio; CI: confidence interval

## Discussion

In our study which looked at infant breastfeeding practices in two cities Central and North Vietnam, the prevalence of exclusive infant breastfeeding in the first six months of life was 14.2%. This prevalence was lower than that found in published data from other regions of Vietnam. The proportion reported by UNICEF in 2019 was 24% for infants less than six months of age [32], and the proportion of infants from birth to five months of age as reported by Multiple Indicator Cluster Surveys was 17% [15]. A recent study conducted in southern Vietnam showed a prevalence of exclusive infant breastfeeding of less than 1% at six months [16]. In comparison to the prevalence of exclusive infant breastfeeding in children younger than six months in East Asia and the Pacific Regions of 31% and in lower-middle-income countries of 61%, our study presented a significantly low prevalence of exclusive breastfeeding [2]. The diversity in study designs, populations, breastfeeding metrics collected, and individual culture’s breastfeeding beliefs may be the reason for the difference in our study’s finding. The method of calculating the breastfeeding rate is an example of this diversity as well. The prevalence of exclusive infant breastfeeding may be identified in some studies based on maternal self-report when the infant is six months old [16] or based on the number of infants less than six months old who received only breast milk in the previous 24 h. Thus, given these confounding study variables, the actual rates of exclusive infant breastfeeding might be lower than what is reported in the published studies.

In our study, mothers who had infants aged over 6 to 30 months were surveyed and the prevalence of exclusively breastfeed infants was defined as the percentage of infants who were fed only with breast milk during the first six months of life. We asked mothers to recall if the child had received breast milk, infant formula, colostrum milk powder, water, vitamin / medicine, fruit juice / honey, and complementary foods when they were under six months of age. Exclusive breastfeeding was identified based on the answer “yes” for the breast milk and “no” for infant formula, colostrum milk powder, water, fruit juice / honey, and complementary foods. Prevalence of exclusive breastfeeding in the current study was based on since-birth recall which differed from those utilized by UNICEF and Multiple Indicator Cluster Surveys which defined exclusive breastfeeding under six months as the percentage of infants 0─5 months of age who were fed exclusively with breast milk during the previous day. Therefore, the low prevalence of exclusive breastfeeding in our study may not actually reflect the declining exclusive infant breastfeeding trend in Vietnam. Several studies compared the used of 24-hour recall with since‐birth recall and showed similar results to ours [33–35]. They found that the use of 24-hour recall overestimated exclusive breastfeeding compared to since‐birth recall [33–35]. A longitudinal prospective study in Sweden, for example, showed a wide discrepancy between the exclusive breastfeeding rate obtained from data based on 24-hour recall and the entire feeding pattern since birth [35]. The difference was over 40% points at two and four months and 9% points at six months.

In our study, we found a high prevalence (63.7%) of infants being fed infant formula in the first six months of their lives. Similarly, a previous study in Vietnam showed infant formula feeding was popular prior to hospital discharge (79.5%) [36]. Further, our results showed a “dose-response” type relationship between the level of maternal exposure to infant formula advertising and exclusive infant breastfeeding. Maternal breastfeeding is affected by advertisements and the widespread availability of breast milk substitutes in Vietnam. The marketing of breast milk substitutes can influence social norms regarding the ease and desirability of infant formula use and undermine a mother’s confidence in breastfeeding her infant [37]. In Vietnam, the government banned the advertising and promotion of breast milk substitutes since 2014 through “Decree 100”. However, infant formula advertising still appears in the media [38]. Breast milk substitutes contribute to increased environmental costs via manufacturing, packaging, distribution, and waste disposal [4]. In Vietnam, a stronger political commitment is required to ensure accountability for marketing these infant formula products.

We identified several other important factors in our study that influenced infant breastfeeding practices. A significant finding from our study is the importance of early skin-to-skin contact. Mothers who had greater than 90 min of skin-to-skin contact and completed the first breastfeeding during skin-to-skin contact were found to have a statistically significant positive association with exclusive infant breastfeeding during the first six months of life. These results are consistent with those of previously published studies [26, 27]. Further, Cesarean section birth is also a known barrier to infant breastfeeding [18]. Currently, the Cesarean section rate in Vietnam is accelerating, and is over 50% of the deliveries in some cities [19, 20], which we also found in the current study (50.4%). Uninterrupted skin-to-skin contact as part of EENC implementation aims to moderate the negative effects of Cesarean section on breastfeeding outcomes. Since November 2014, and in accordance with the Ministry of Health Hospital Policy Directive 4673 (EENC implementation for uncomplicated Cesarean section births), policies were implemented in hospitals throughout Vietnam to follow this directive [13]. According to the protocol, the baby is to be placed on the mother’s chest area immediately after delivery to establish skin-to-skin contact, while the remaining procedures for a Cesarean operation are practiced as usual. When possible, regional anaesthesia is recommended instead of general anaesthesia because it allow mothers to support their infant in maintaining skin-to-skin contact. For mothers who receive general anesthesia, the anaesthetist must carefully monitor to ensure mother’s pulse oximetry is > 95%, and one staff member is responsible for monitoring and supporting the newborn on their mother’s chest. After delivery, the infant and mother are transferred to the recovery room in the skin-to-skin contact position. Uninterrupted skin-to-skin contact is to be maintained for at least 90 min in the recovery room and until the completion of first breastfeeding. As shown in Tables 2, 87.3% of mothers in our study experienced skin-to-skin contact with their infant right after birth. The high rate of skin-to-skin contact practice in Vietnam as well as the positive effects of duration of skin-to-skin contact on exclusive infant breastfeeding during the first six months suggest that skin-to-skin contact and early initiation of infant breastfeeding, as part of EENC implementation, appear to mitigate the negative effects of Cesarean section birth on exclusive infant breastfeeding.

We also found that a mother’s prenatal intention to exclusively breastfeed after delivery was associated with a higher probability of exclusive infant breastfeeding for the first six months of the infant’s life. Our current study confirms similar previous findings in the literature [39]. Despite a high proportion of mothers (74.2%), intending to exclusively breastfeed, non-breastfeeding practices such as discarding colostrum and using colostrum milk powder, giving water and honey to the infant, and feeding the children with extra infant formula whenever they cry because of the belief in mother’s milk supply being insufficient remain common practices in Vietnam [25]. If mothers can have a better understanding about the benefits of infant breastfeeding, and of the idea of “exclusive breastfeeding”, as well as have positive beliefs and confidence in their breastfeeding by receiving support from family members and healthcare workers, they are more likely to exclusively breastfeed their infants [40].

Our study has several limitations. First, the relationship between predictors and exclusive infant breastfeeding was not causally related because of the disadvantage of a cross-sectional study design. Second, maternal recall bias is another concern when interpreting the results. Third, there may exist a selection bias as we did not survey women who brought their infants to a private clinic / hospital for vaccination results due to the difficulties of getting data collection approval from private settings in the context of the COVID-19 pandemic. However, as most of the children in Vietnam are vaccinated in public settings, data from our sample may not be far from the entire population. Further, we did not collect breastfeeding data on infants who were brought to CHCs by fathers or grandparents. Nevertheless, our current study on the prevalence of exclusive breastfeeding and associated factors which influence breastfeeding decisions and practices could be a useful source for stakeholders and policymakers to develop an appropriate and effective intervention to promote breastfeeding, especially in low–middle income countries like Vietnam.

## Conclusion

In our current study, we found the prevalence of exclusive infant breastfeeding for the first six months of life in Vietnam is low. Our study findings regarding factors associated with an increased probability of exclusive infant breastfeeding should be utilized to develop measures to protect, promote, and support infant breastfeeding. In particular, promoting early maternal-infant skin-to-skin contact of more than 90 min duration and completion of first breastfeeding during skin-to-skin contact should be encouraged. A greater political commitment to hospital practices and infant formula milk marketing is needed to reduce the mother’s prenatal exposure to infant formula advertising.

## Data Availability

The datasets used and / or analysed during the current study are available from the corresponding author on reasonable request.

## List of abbreviations

CHC: Community Health Centre
EENC: Early Essential Newborn Care
WHO: World Health Organization
